# Retrospective use of whole genome sequencing to better understand an outbreak of *Salmonella enterica* serovar Mbandaka in New South Wales, Australia

**DOI:** 10.5365/wpsar.2017.8.4.008

**Published:** 2018-04-23

**Authors:** Cassia Lindsay, James Flint, Kim Lilly, Kirsty Hope, Qinning Wang, Peter Howard, Vitali Sintchenko, David N Durrheim

**Affiliations:** a Hunter New England Health, New South Wales, Australia.; b Health Protection NSW, New South Wales, Australia.; c Centre for Infectious Diseases and Microbiology, Institute of Clinical Pathology and Medical Research, Westmead Hospital, New South Wales, Australia.

## Abstract

**Introduction:**

*Salmonella enterica* serovar Mbandaka is an infrequent cause of salmonellosis in New South Wales (NSW) with an average of 17 cases reported annually. This study examined the added value of whole genome sequencing (WGS) for investigating a non-point source outbreak of *Salmonella* ser. Mbandaka with limited geographical spread.

**Methods:**

In February 2016, an increase in *Salmonella* ser. Mbandaka was noted in New South Wales, and an investigation was initiated. A WGS study was conducted three months after the initial investigation, analysing the outbreak *Salmonella* ser. Mbandaka isolates along with 17 human and non-human reference strains from 2010 to 2015.

**Results:**

WGS analysis distinguished the original outbreak cases (*n* = 29) into two main clusters: Cluster A (*n* = 11) and Cluster B (*n* = 6); there were also 12 sporadic cases. Reanalysis of food consumption histories of cases by WGS cluster provided additional specificity when assessing associations.

**Discussion:**

WGS has been widely acknowledged as a promising high-resolution typing tool for enteric pathogens. This study was one of the first to apply WGS to a geographically limited cluster of salmonellosis in Australia. WGS clearly distinguished the outbreak cases into distinct clusters, demonstrating its potential value for use in real time to support non-point source foodborne disease outbreaks of limited geographical spread.

## Introduction

*Salmonella enterica* serovar Mbandaka is a relatively uncommon *Salmonella* serovar in New South Wales (NSW) with an average of 17 cases notified per year over the past 10 years. (*1*) *Salmonella* ser. Mbandaka cases reported in Australia have been acquired locally and overseas in India, Africa, Indonesia, Mexico and China. (*2*) In Australia, *Salmonella* ser. Mbandaka has been isolated from foods such as chicken, peanut butter, turkey meat and curry powder. (*2*) Whole genome sequencing (WGS) is a high-resolution typing method that can help foodborne disease investigators distinguish outbreak cases from non-outbreak cases. (*3*) WGS has been used for public health surveillance in the United States of America, United Kingdom of Great Britain and Northern Ireland, and the European Union. (*4*-*6*) In Australia, several jurisdictional reference laboratories are developing WGS capacity and evaluating its utility for routine surveillance of enteric pathogens. (*7*) This study examined the potential added value of WGS in assisting investigators identify the source of a community outbreak of *Salmonella* ser. Mbandaka with limited geographical spread.

## Methods

In February 2016, an increase in *Salmonella* ser. Mbandaka notifications was noted in the Hunter New England and Central Coast local health districts of NSW. A confirmed case was defined as any resident or visitor to NSW with laboratory-confirmed *Salmonella* ser. Mbandaka infection and symptom onset from 1 January 2016 to 30 April 2016. Individuals meeting the case definition were interviewed by phone, beginning 22 February 2016, using a standard *Salmonella* hypothesis-generating questionnaire to collect demographic, clinical and risk factor information, including travel and food consumption histories during the seven days before illness onset. For reference, data from a 2016 Victorian Food Consumption study were used to provide expected food consumption frequencies in a healthy population. This data set contains seven-day food consumption histories of approximately 500 randomly selected healthy individuals in Victoria from January to April 2016, the same time period as the *Salmonella* ser. Mbandaka outbreak. The Victoria data set was used because no equivalent NSW data set exists. Food consumption frequencies of outbreak cases were compared to those from the Victorian Food Consumption study using binomial probability.

Illness onset dates were documented during case interviews or estimated based on specimen collection dates, using the average incubation period from all other cases, for cases lost to follow-up. The WGS study was conducted retrospectively three months after the initial outbreak investigation, analysing the *Salmonella* ser. Mbandaka isolates associated with this outbreak and comparing them with 10 human strains from 2010 to 2015 and six non-human isolates from 2012 to 2015 (primarily egg farm swabs from the NSW Food Authority). WGS was conducted by the NSW Enteric Reference Laboratory, Institute of Clinical Pathology and Medical Research, NSW Health Pathology. For WGS, the DNA was extracted and purified using a DNeasy Blood and Tissue Kit (Qiagen, Hilden, Germany) according to the manufacturer’s instructions. DNA quantities were estimated using the Qubit dsDNA HS Assay Kit and the Qubit Fluorometer (Thermo Fisher Scientific, Waltham, MA, USA) according to the manufacturer’s instructions. For each purified DNA sample, a 100 bp library was prepared using the NexteraXT kit (Illumina, Inc., San Diego, CA, USA), then pooled and sequenced on the NextSeq500 platform (Illumina). FastQ files were imported into CLC Genomics Workbench v 7.0 (CLC bio, Aarhus, Denmark); reads were trimmed to remove Nextera transposase adaptor sequences and then mapped to the reference genome *Salmonella* ser. Mbandaka str. ATCC 51958 (NCBI GenBank accession: CP019183.1).

Clusters were identified based on sequence similarity between *Salmonella* ser. Mbandaka genomes using single nucleotide polymorphism (SNP) analysis. The SNP phylogenetic tree was generated through the concentrated SNP alignments using MEGA7 sequence analysis software (https://www.megasoftware.net) with a bootstrap value at 100. (*8*)

The food consumption histories were reanalysed based on the clusters identified by WGS and compared to the data from the 2016 Victorian Food Consumption study. Data were entered and analysed in EpiInfo (Version 7) and Microsoft Excel.

### Ethics statement

This work was part of an outbreak investigation and did not require ethical review and oversight by a Human Research Ethics Committee.

## Results

**Fig. 1:**
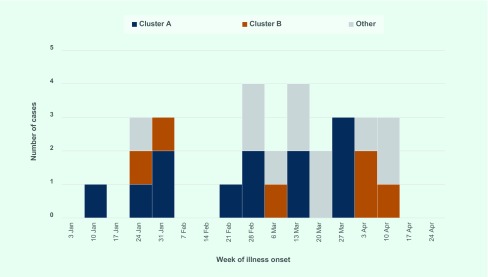
Confirmed cases (*n* = 29) of *Salmonella* ser. Mbandaka in New South Wales by cluster and week of illness onset, 3 January to 30 April 2016

From 1 January 2016 to 30 April 2016, 29 cases of salmonellosis caused by *Salmonella* ser. Mbandaka were notified. The epidemic curve of cases investigated as part of this outbreak is shown in **Fig. 1**. Illness onset dates ranged from 15 January to 14 April 2016. Seven case patients were hospitalized and no patients died. Patients were aged from 1 to 89 years with a median age of 48 years, 14 (48%) lived in the Hunter New England Local Health District, 16 (55%) were male and three (10%) were of Aboriginal origin. Commonly reported symptoms included diarrhoea (*n* = 21, 95%), lethargy (*n* = 17, 85%), abdominal pain (*n* = 14, 64%), fever (*n* = 13, 62%) and vomiting (*n* = 12, 55%). Symptoms continued for 1–10 days (median five days).

The initial (pre-WGS) investigation did not identify any common eating establishments or shopping venues among cases. Processed cheese was identified to have a higher-than-expected consumption frequency among cases; it was consumed by 64% of cases when the expected consumption frequencies in a healthy population was 22% (binomial probability [*P* = 0.0008]). However, on closer analysis, several different brands of processed cheese and places of purchase were indicated, and in the absence of additional cases, no food safety investigation was initiated. Other foods with a higher-than-expected consumption frequency included watermelon (63%, *P* = 0.0341, *P* = 0.04), onion (69%, *P* = 0.0574, *P* = 0.07) and green capsicum (53%, *P* = 0.0599, *P* = 0.07).

**Fig. 2:**
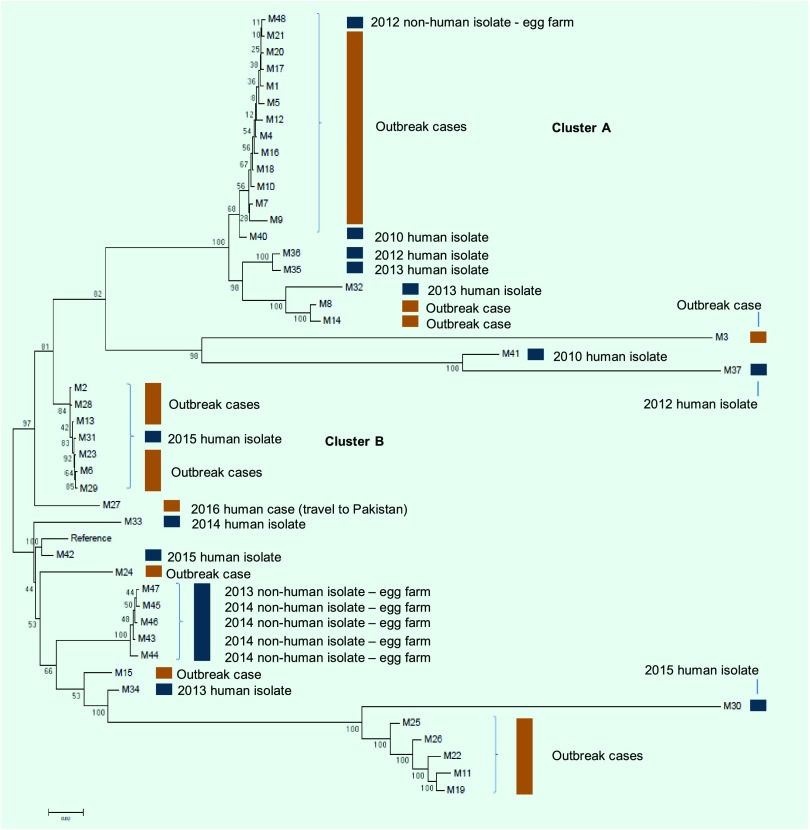
Phylogenetic tree generated from whole-genome SNPs for outbreak and non-outbreak cases of *Salmonella* ser. Mbandaka in NSW

WGS analysis distinguished the original outbreak cases into two main clusters: Cluster A, which included 11 cases with an SNP distance between 12 and 82, and Cluster B, which included six cases with an SNP distance between 10 and 25 (**Fig. 2**). In addition to the two key clusters, WGS identified smaller clusters and several sporadic cases. The food consumption frequencies re-analysed by the two key clusters are shown in **Table 1**. The consumption of processed cheese among cases in Cluster A increased to 89% (*P* < 0.0001) and decreased in Cluster B to 33%. (*P* = 0.5254) when compared to all cases (**Table 1**).

**Table 1:** Food consumption frequencies among all cases and by key clusters identified by WGS

Food Item	All cases			WGS – Cluster A			WGS – Cluster B			Reference*		
	Ate food	Total	%	Ate food	Total	%	Ate food	Total	%	Ate food	Total	%
**Tomato**	**11**	**13**	**85%**	**9**	**9**	**100%**	**2**	**3**	**67%**	**497**	**665**	**75%**
**Carrot**	**12**	**16**	**75%**	**9**	**10**	**90%**	**3**	**4**	**75%**	**534**	**662**	**81%**
**Potato**	**10**	**14**	**71%**	**9**	**9**	**100%**	**1**	**3**	33%**	**546**	**667**	**82%**
**Onion**	**11**	**16**	**69%**	**8**	**10**	80%**	**3**	**4**	**75%**	**307**	**666**	**46%**
**Chicken pieces**	**11**	**16**	**69%**	**6**	**10**	**60%**	**3**	**4**	**75%**	**406**	**664**	**61%**
**Black pepper**	**11**	**16**	**69%**	**8**	**10**	**80%**	**3**	**4**	**75%**	**427**	**666**	**64%**
**Processed cheese**	**9**	**14**	64%**	**8**	**9**	89%**	**1**	**3**	**33%**	**149**	**665**	**22%**
**Free range eggs**	**7**	**11**	**64%**	**4**	**6**	**67%**	**2**	**3**	**67%**	**291**	**446**	**65%**
**Eggs (any)**	**7**	**11**	**64%**	**7**	**9**	**78%**	**3**	**4**	**75%**	**446**	**665**	**67%**
**Watermelon**	**10**	**16**	63%**	**8**	**10**	80%**	**2**	**4**	**50%**	**245**	**667**	**37%**
**Apple**	**10**	**16**	**63%**	**6**	**10**	**60%**	**3**	**4**	**75%**	**446**	**667**	**67%**
**Banana**	**10**	**16**	**63%**	**6**	**10**	**60%**	**3**	**4**	**75%**	**464**	**667**	**70%**
**Beef mince**	**8**	**14**	**57%**	**7**	**8**	88%**	**1**	**4**	**25%**	**332**	**663**	**50%**
**Green capsicum**	**8**	**15**	**53%**	**6**	**9**	66%**	**2**	**4**	**50%**	**209**	**667**	**31%**
**Grapes**	**8**	**15**	**53%**	**4**	**9**	**44%**	**3**	**4**	**75%**	**375**	**666**	**56%**
**Red capsicum**	**8**	**15**	**53%**	**6**	**9**	**67%**	**2**	**4**	**50%**	**304**	**667**	**46%**
**Broccoli**	**8**	**15**	**53%**	**6**	**9**	**67%**	**2**	**4**	**50%**	**348**	**665**	**52%**
**Cucumber**	**8**	**15**	**53%**	**5**	**9**	**56%**	**3**	**4**	**75%**	**383**	**665**	**58%**

* Reference = data from 2016 Victorian Food Consumption study

** Difference from reference statistically significant (< 0.05)

## Discussion

Internationally, WGS is increasingly being used for enhanced foodborne disease surveillance and response due to its discrimination power for typing cases and tracing infection sources and its similar turnaround times to other laboratory techniques. (*5*, *6*) WGS is also being used to understand disease transmission pathways and determinates of transmission, monitor pathogen evolution and adaptation, identify infections with epidemic potential and refine control strategies. (*9*)

In the United States, WGS is replacing pulsed-field gel electrophoresis for subtyping foodborne pathogens for outbreak surveillance. (*10*) WGS of foodborne pathogens is used for regulatory purposes by the US Food and Drug Administration (*5*) and has proven valuable in outbreak investigations for differentiating sources of contamination. (*11*, *12*) In European Union countries and in the United Kingdom, WGS is increasingly being used for foodborne disease outbreak investigations and national surveillance of infectious diseases. (*4*, *6*) In Australia, WGS is acknowledged as a promising typing alternative; however, it is not yet in widespread use due to limitations in standardized quality control and data interpretation, cost and infrastructure. (*13*, *14*) WGS is being piloted by OzFoodNet, an Australian Department of Health foodborne disease surveillance and response network, and has been successfully applied in multijurisdictional foodborne disease outbreaks and for routine surveillance of *Listeria monocytogenes*. (*3*, *15*)

This *Salmonella* ser. Mbandaka study was one of the first in Australia to apply WGS to a geographically limited cluster of *Salmonella*. Although the WGS was not conducted in real time, its potential to support an outbreak investigation was demonstrated. WGS was able to differentiate the outbreak cases of *Salmonella* ser. Mbandaka into distinct clusters and sporadic cases. Analysis of food consumption histories based on phylogenetic cluster suggests two concurrent outbreaks of *Salmonella* ser. Mbandaka may have occurred in NSW. If WGS had been conducted in real time, affected individuals would have been reinterviewed to collect additional details on food items of interest and further analysis conducted. Our findings support an earlier study in NSW that applied WGS retrospectively to five epidemiologically confirmed community outbreaks of *Salmonella enterica* serovar Typhimurium and found that WGS significantly increased the resolution of investigations. Their study also found that for one of the outbreaks, the food source was contaminated with more than one strain of *Salmonella* ser. Typhimurium, highlighting the need to assess both laboratory and epidemiological information during an investigation.

Data from the Victorian Food Consumption study allowed investigators to estimate expected food consumption frequencies in a healthy population and, using binomial probabilities, compare them to the food consumption frequencies among the outbreak cases. This method allows for rapid hypothesis generation to guide further environmental and epidemiological investigations. The absence of an equivalent NSW food consumption data set was a limitation of this study. It was assumed that food consumption habits and available foods in Victoria and NSW were similar enough to permit hypothesis generation. Given the potential for differences in food habits or food availability between the two populations, the associations derived need to be interpreted with caution and used for hypothesis generating rather than testing. The rapid development in advanced laboratory tools also presents challenges for public health practitioners. As public health reference laboratories have been adopting WGS, clinical laboratories are increasingly relying on culture-independent multiplexed molecular panels to test stool specimens for enteric pathogens. (*16*) The move away from culturing enteric pathogens will reduce the number of isolates available for typing by WGS or other culture-dependent typing methods. In response, scientists are working to develop metagenomic sequencing-based tools to characterize stool specimens without the need for culture. (*7*, *17*) As these developments continue to evolve, health practitioners will need to understand how they will impact surveillance systems, outbreak detection and response activities.

In conclusion, this study highlighted the potential value of WGS in supporting epidemiologists to investigate a relatively small, non-point source foodborne disease outbreak in a community. If conducted in real time, WGS could have assisted with potential source detection to guide further investigations and to aid control efforts. The continued application of WGS to support foodborne disease outbreak investigations in Australia will contribute to a global understanding of its potential to control outbreaks in a more timely and efficient manner.
